# "Good idea but not feasible" – the views of decision makers and stakeholders towards strategies for better palliative care in Germany: a representative survey

**DOI:** 10.1186/1472-684X-8-10

**Published:** 2009-07-22

**Authors:** Sara Lena Lueckmann, Mareike Behmann, Susanne Bisson, Nils Schneider

**Affiliations:** 1 Hannover Medical School, Centre for Public Health, Institute for Epidemiology, Social Medicine and Health System Research, Carl-Neuberg-Str.1, 30625 Hannover, Germany; 2 Hannover Medical School, Centre for Public Health, Endowed Chair Prevention and Rehabilitation in Health System and Health Services Research, Carl-Neuberg-Str.1, 30625 Hannover, Germany

## Abstract

**Background:**

Statements on potential measures to improve palliative care in Germany predominantly reflect the points of view of experts from specialized palliative care organizations. By contrast, relatively little is known about the views of representatives of organizations and institutions that do not explicitly specialize in palliative care, but are involved to a relevant extent in the decision-making and policy-making processes. Therefore, for the first time in Germany, we carried out a representative study of the attitudes of a broad range of different stakeholders acting at the national or state level of the health care system.

**Methods:**

442 organizations and institutions were included and grouped as follows: patient organizations, nursing organizations, medical associations, specialized palliative care organizations, political institutions, health insurance funds and others. Using a standardized questionnaire, the participants were asked to rate their agreement with the World Health Organization's definition of palliative care (five-point scale: 1 = completely agree, 5 = completely disagree) and to evaluate 18 pre-selected improvement measures with regard to their general meaningfulness and the feasibility of their introduction into the German health care system (two-point scale: 1 = good, 2 = poor).

**Results:**

The response rate was 67%. Overall, the acceptance of the aims of palliative care in the WHO definition was strong. However, the level of agreement among health insurance funds' representatives was significantly less than that among representatives of the palliative care organizations. All the improvement measures selected for evaluation were rated significantly higher in respect of their meaningfulness than of their feasibility in Germany. In detail, the meaningfulness of 16 measures was evaluated positively (70–100% participants chose the answer "good"); for six of these measures feasibility was evaluated negatively (0–30% "good"), while for the remaining ten measures feasibility was evaluated inconsistently (31–69% "good").

**Conclusion:**

The reason why potentially meaningful improvement measures are considered to be not very feasible in Germany may be the existence of barriers resulting from the high degree of fragmentation of health care provision and responsibilities. In overcoming these barriers and further improving palliative care it may be helpful that the basic understanding of the palliative care approach seems to be quite homogenous among the different groups.

## Background

Palliative care is an approach that improves the quality of life of patients and their families facing the problems associated with life-threatening illness [1]. Despite some advances in recent years, palliative care is still an underdeveloped field in many countries with health systems that are in other respects highly developed [2-5].

In Germany there is a widespread undersupply of specialist palliative care both for inpatients and in particular for outpatients. At present, there are about 330 hospices and palliative care units nationwide, with a total of about 2,800 beds (i.e. about 34 beds per million inhabitants), and about 60 palliative care teams in all (for 80 million inhabitants). Moreover, there are considerable regional differences, with very well developed services in some urban areas and extensive gaps in the periphery [6-8]. Besides, there are problematical deficits in the education and the advanced training of all health care professions regarding palliative care; for example, the subject of palliative medicine is not compulsory in medical schools, and is therefore only taught here and there [9].

However, in recent years palliative care has achieved increasing recognition among the public and in political circles. For example, a big step forming part of the recent (2007) health care reform was the legal introduction of specialist outpatient palliative care (SAPV), i.e. outpatient care delivered by palliative care teams [10]. So far, the realization of SAPV is very problematic; e.g., there are no uniform standards regarding the staffing and structural requirements of palliative care teams, and their cooperation with other players (especially family doctors, nursing services, specialist physicians in other medical disciplines) is not consistently regulated [11].

It has become clear that the further development of palliative care is a major social and political task [6,7,9,12]. In this context it is important to consider that the political decision-making processes in Germany are influenced by numerous lobby groups, organizations and institutions, due to the legal principles of federalism, the self-governing status of medical services and health insurance funds and the separation between outpatient and inpatient services [13,14]. Evaluating the attitudes of the different stakeholders is therefore an important aim of health service research.

So far, statements on potential measures to improve palliative care have mainly reflected the points of view of experts from specialized palliative care and hospice organizations and institutions. By contrast, very little is known about the views of representatives of organizations and institutions that do not explicitly specialize in palliative care, but are involved to a relevant extent in the decision-making and policy-making processes [15]. These are, for example, the various medical and nursing associations, health insurance funds, patients' organizations, and government and regulatory authorities and political parties (these last mentioned being referred to collectively below as "political institutions").

For the first time in Germany we wanted to representatively evaluate the views of a wide range of different stakeholders, acting at the meso and macro level of the health system, concerning selected improvement measures for palliative care. The study was part of a larger research project aimed at developing public health targets for palliative care in Germany. The conceptual framework and design of the overall project are published elsewhere [16].

## Methods

### Ethics approval

The Ethics Committee of Hannover Medical School approved the study (letter of 26/02/07).

### Sample

For the recruitment of the study population a snowball sampling approach was used. First of all, the members of the study group compiled a list of institutions and organizations based on their respective individual experience (e.g. NSCH is a lecturer in public health and health system research as well as a consultant in family medicine and palliative medicine). The list was supplemented out of the literature and by internet research, as well as by discussion rounds with further public health specialists in our department.

The main inclusion criteria for the organizations were:

- being involved in the provision, financing and/or political organization of health care;

- being associated with a lobby group of health care professionals, patients, politicians and/or health insurance funds;

- being active at the state or national level of the German health care system.

This led to our including of 366 institutions and organizations in the first round of the survey. In order to identify further relevant players, we asked the participants initially included: *"An important aim of this study is to assess the views of all relevant stakeholders within the context of palliative and end-of-life care in Germany. We would therefore like to ask you to write down the names of other players, institutions and organizations which in your opinion should also be questioned." *In this way we identified a further 76 stakeholders that had not already been included in the first round.

In the end, 442 organizations and institutions were included and grouped (Table 1). The groups had been predefined when the project was designed [16]: A: patient organizations, B: medical associations, C: nursing organizations, D: health insurance funds, E: political institutions, F: specialized palliative care organizations and G: others. The group "others" is included in the outcome of analyses for all groups, but not in the comparison of the groups because of its high level of heterogeneity.

**Table 1:** Group sizes and characteristics of organizations

**ID**	**Group**	**group size ** **sample ** **(contacted)**	**response ** **rate**	**final group ** **size ** **(analyzed)**	**proportion** **(of all ** **analyzed)**	**characteristics** ** (examples)**
	all groups	442	67%	301	100%	
A	patient organizations	42	57%	24	8%	umbrella organizations of self-help groups and senior citizens interest groups, federation of German consumer centres
B	medical associations	100	78%	79	26%	scientific medical societies, associations of statutory health insurance physicians on federal and national level
C	nursing organizations	22	77%	17	6%	umbrella organizations of nursing care, federal working commitee of nursing
D	health insurance funds	82	63%	55	18%	associations of health insurance funds on federal and national level, umbrella organizations of health insurance funds
E	political institutions	45	49%	24	8%	state and federal ministries of health, health-care policy spokespeople of the different political parties
F	specialized palliative care	54	65%	39	13%	associations and umbrella organizations of palliative and hospice care on federal and national level
G	others*	97	72%	63	21%	charity and clerical institutions, associations for physiotherapy, federal joint committee

* not included in the comparison of the groups because of the strong heterogeneity

### Procedure

The 366 institutions approached in the first round were sent a letter of advice in April 2008 in which they were informed about the study and invited to participate. Initially, the letter was addressed to the heads of the institutions and organizations. The addressees were asked to forward the questionnaire to another person within the organization if they themselves did not feel responsible or qualified enough in palliative care.

One week after the information letter we mailed the questionnaires together with further information, instructions and a stamped addressed envelope. Four weeks later all organizations received a postcard of thanks which included the request to fill in the questionnaire if they had not already done so. Another five weeks later, those organizations that had not yet answered received a further reminder with one more questionnaire.

The additional 76 stakeholders identified later were addressed in the same way. The survey was carried out from April to August 2008.

### Instrument

We developed a standardized questionnaire. To study the participants' basic understanding of the palliative care approach, we sought to identify the acceptance of selected aspects from the World Health Organization's definition of palliative care [1] using a five-point scale (1 = completely agree, 2 = agree, 3 = undecided, 4 = disagree, 5 = completely disagree).

Additionally, the questionnaire contained 18 items that presented potential measures aiming to improve palliative care in Germany. These measures were selected on the basis of literature reviews and of our own pre-studies [e.g. [15]]. Their potential relevance to the development of public health targets for palliative care in Germany [16] was also taken in consideration. The participants were asked to assess the measures with regard to their meaningfulness in general and also to their feasibility in Germany. The answers could be given using a two-point scale (1 = good, 2 = poor).

In order to evaluate the comprehensibility and practicability of the questionnaire, a cognitive pre-test was carried out with 13 participants not included in the study. The pre-test participants had to fill in the questionnaire and reply to additional questions focusing on their understanding of the questionnaire (probing method). Furthermore, the participants could ask about the tenor of the questions. After seven pre-tests we interrupted the testing since we detected some room for improvement. After revising the questionnaire we carried out another six pre-tests resulting in some further minor revisions.

### Statistics

Data were recorded with MS Access (2003) and transferred to SPSS for Windows 16.0 for statistical analysis. Data are reported descriptively as frequencies in percent. The McNemar test was used to analyze the differences between the evaluations of the meaningfulness and the feasibility of the improvement measures; the chi-square test was used to examine proportional differences between the groups.

The evaluation of the World Health Organization's definition of palliative care on a five-point scale was reduced to a three-point scale to avoid too small cell frequencies. Because of the asymmetrical distribution we decided to combine the answer choices 3–5 (3 = undecided, 4 = disagree, 5 = completely disagree) into one (3 = undecided, disagree and completely disagree).

We defined the assessment of measures as positive when = 70% of participants chose the answer "good", and as negative when = 30% chose the answer "good". The answers were assessed as being inconsistent when 31–69% of participants chose the answer "good". P < .05 was considered statistically significant.

## Results

295 stakeholders replied to the questionnaire. Overall, the response rate was 67%. The response rate was best for the group of medical associations (78%, n = 78) and worst for the group of political institutions (49%, n = 22). Three organizations asked for more than one questionnaire or copied the original because representatives from different departments within the organization wanted to participate. In these three exceptional cases we accepted this. As a result, the group size was changed for three groups: medical associations (n+1), health insurance funds (n+3) and political institutions (n+2). In the end, we were able to analyze a total of 301 questionnaires (Table 1).

The demographic data of the respondents are shown in Table 2. The respondents were between 25 and 73 years of age (mean 51), 62% were male and 40.7% had studied medicine. 26.8% of the questionnaires were answered by a member of the board and 24.4% by a head of department. 53% of the participants had been employed in the institutions for more than 10 years.

**Table 2:** Characteristics of the respondents

**demographic data**	**%**	**characteristics**
age		51 ± 9.0 (Range: 25–73 years)
	26.8	till 45 (n = 75)
	45.0	46–55 (n = 126)
	28.2	56+ (n = 79)

sex	62.0	male (n = 184)
	38.0	female (n = 114)

nationality	99.0	German (n = 293)

professional background*	40.7	medicine (n = 120)
	12.2	economics (n = 36)
	9.8	social science (n = 29)
	13.2	nursing (n = 39)
	8.5	others (n = 25)

position within the organization/institution*	26.8	member of the board (n = 79)
	24.4	head of department (n = 72)
	19.7	business manager (n = 58)
	16.6	consultant (n = 49)
	4.7	volunteer (n = 14)

time of employment in the institution	53.0	> 10 years (n = 157)
	22.3	6–10 years (n = 66)
	22.0	1–5 years (n = 65)
	2.7	<1 year (n = 8)

religion	40.6	catholic (n = 119)
	34.8	protestant (n = 102)
	23.2	undenominational (n = 68)
	1.4	other religion (n = 4)

* multiple answers possible

The groups differed significantly in distribution of sex (p = .004): in the groups patient organizations (A), nursing organizations (C), political institutions (E) and specialized palliative care (F) there were more women, whereas in the groups medical associations (B) and health insurance funds (D) there were more men answering the questionnaire. There was no significant difference between the groups in the distribution of age (p = .12).

### Understanding of palliative care

The acceptance of the seven aspects of the World Health Organization's definition of palliative care that were asked about was strong [see Additional file 1]. The most pronounced agreement was found to the statement *"In my opinion it is part of appropriate palliative care to provide relief from pain and other distressing symptoms": *99% (n = 298) of the respondents showed complete or predominant agreement. Relatively less pronounced agreement was found to the statement *"In my opinion it is part of appropriate palliative care to intend neither to hasten nor to postpone death": *72% (n = 213) of the respondents showed complete or predominant agreement.

Comparing the six groups with regard to their weighting of the aspects of the World Health Organization's definition we found statistically significant differences with regard to six out of seven statements [see Additional file 1]. By way of example, the statement *"... to integrate the psychological and spiritual aspects of patient care" *was most positively assessed by the representatives of specialized palliative care organizations (97.4% agreed completely) and least positively by the health insurance funds' representatives, of whom only 49.1% completely agreed. The statement *"... to offer a support system to help the family cope during the patient's illness" *was most positively assessed by the representatives of specialized palliative care organizations (79.5% agreed completely) and least positively by the health insurance funds' representatives (of whom only 20% completely agreed).

All in all, in contrast to the specialized palliative care organizations' representatives, the health insurance funds' representatives agreed considerably less with the WHO definition of palliative care.

### Assessment of improvement measures

Overall, the feasibility of potential improvement measures in Germany was evaluated considerably less positively than their meaningfulness [see Additional file 2]. The discrepancy between meaningfulness and feasibility is significant for all 18 measures that were presented (p < .001).

In detail, the meaningfulness of 16 measures was evaluated as being positive (= 70% "good"), whereas the feasibility of six out of these 16 measures was evaluated as being negative (= 30% "good"). This concerns all measures regarding the topic *caring time *(items 5–8) as well as the measures of *nursing home physicians *(item 14) and *academic centres for research *(item 17). For these six measures the discrepancy is the greatest. The feasibility of the remaining ten (out of these 16) measures was evaluated inconsistently (31–69% "good").

Both the meaningfulness and the feasibility of the measure "*Palliative care patients are completely exempted from additional payments for drugs, cures and aids" *(item 12) were evaluated inconsistently.

In the case of the measure *"Case managers organize and coordinate the care of palliative care patients *(item 15)", was the meaningfulness evaluated inconsistently while the feasibility was evaluated negatively.

Comparing the six groups with regard to their rating of the meaningfulness and feasibility of the measures we found statistically significant differences for some statements [see Additional file 2]:

- Representatives from patient organizations and/or specialized palliative care evaluated the meaningfulness of six measures (items 2, 3, 8, 10, 11, 17), namely those concerning the topics *education *and *training*, as well as *availability of 24-7 regional palliative care services *and *establishment of publicly funded academic centres for research in palliative care*, more highly than the other groups did.

- Nursing organizations evaluated the meaningfulness of three measures more highly than the other groups did: *palliative care patients are completely exempted from additional payments for drugs, cures and aids *(item 12), *regular availability of nursing home physicians with specialized training in palliative care *(item 14), *case managers organize and coordinate the care of palliative care patients *(item 15).

- Health insurance funds evaluated the feasibility of *compulsory training in palliative medicine for medical students *(item 2) more highly than the other groups did.

To sum up, the representatives of patient, nursing and specialized palliative care organizations evaluated the meaningfulness of the proposed improvement measures for palliative care in Germany considerably better than the other groups did. However, the feasibility of all improvement measures received a significantly worse assessment in all cases compared to the assessment of their meaningfulness.

## Discussion

This study is, to our knowledge, the first one that gives representative insights into the views of a broad range of decision-makers and lobby groups at the meso and macro level of the German health system with regard to various improvements in palliative care. The results broaden the perspectives and point to possible strategies aimed at promoting further scientific and political development in the field. For example, they might be integrated into the *"charter process for the care of very severely ill and dying people" *that has recently been initiated by the German Association for Palliative Medicine (DGP), the German Hospice and Palliative Organization (DHPV) and the German Medical Association (BÄK) [17]. The aims of this charter are to promote dialogue between all those concerned and the involvement of society with the subject, to provide guidance for the direction of future developments and to reach agreement on common objectives and actions. The charter relates above all to issues of social policy, especially to ethical and legal issues, the further development of care structures, questions of initial and in-service training in the various professions concerned and issues relating to research.

The response rate (67%) was good considering the fact that most of the stakeholders included in the study, while involved in the topic to a greater or lesser extent, were not specialists in it. Therefore, most of the participants presumably had no overriding professional interests in palliative care. The response rate was better than in previous surveys on palliative care in Germany which involved health professionals [16]. This affirms the high level of interest in palliative care and end-of-life topics in society.

We found the basic acceptance of the aims of palliative care to be strong among the stakeholders, which might be helpful to the development of the charter process mentioned above [17]. However, the agreement of the health insurance funds' representatives to the majority of the palliative care issues presented was significantly lower than that of the palliative care organizations' representatives. This may result from a different awareness due to the usual absence of clinical experience on the part of health insurance funds' representatives, and also from the different aims and priorities due to the professional responsibilities and tasks in the health care system. The conclusion may be drawn that it is important to respect differing views, to learn from each other and to increase awareness of the specific palliative care approach among those who are not specialists.

Altogether there was consensus among the stakeholders about the meaningfulness of the measures for improvement presented. Compulsory training of health professionals in palliative care (items 1–3) met with the participants' greatest approval. This is not surprising as training deficits are among the major problems in palliative and end-of-life care in Germany despite advances in recent years, e.g. the implementation of the optional qualification in palliative medicine for physicians [6,9,18]. For example, at many universities palliative care is still not part of the curriculum and thus not regularly taught to medical students [9]. In contrast to its meaningfulness, the feasibility of compulsory training in palliative care (item 2) was given a far worse assessment. This is at first glance surprising, because it seems unclear why it should be so difficult to integrate palliative care training into the curricula for medical students. Maybe the already crowded curricula make it difficult to add further content. However, it has to be asked why this seems to be so difficult in Germany whereas it has been realized in other countries, e.g. France or Norway [6].

This brings up the key issue arising from the results: The *feasibility *of all measures was given a significantly worse assessment than their *meaningfulness*. Moreover, the *meaningfulness *of most measures (16 out of 18) was assessed positively whereas the *feasibility *of the measures in Germany was evaluated negatively (7 out of 18) or inconsistently (11 out of 18). Why does it appear to be so difficult to implement substantial changes in the German health system?

The German health system is based on local decision-making and the democratic legitimization of self-governing structures which are safeguards against unwanted government interference [19]. On the other hand, numerous players and lobby groups often represent different, conflicting interests; they may attempt to steer political developments in one particular direction or another in order to achieve advantages for their own pressure group. This complicates the political decision-making processes and may lead to substantial changes being implementable only with difficulty, since it is almost always the case that there are different lobby groups trying to promote different interests. As politicians are dependent on winning votes, lobby groups exercise great influence on voters and elections at local, state or federal level take place all the time in Germany and frequently lead to unstable political majorities, the result may be a certain degree of log-jam where major reforms in the field of health care are concerned.

For example, one serious weakness in the German system is the traditional fragmentation of care across the different health care sectors (e.g. inpatient and outpatient care, rehabilitation), which has been addressed by several recent reforms but has still not been satisfactorily overcome, resulting in serious difficulties in realizing substantial structural changes [13,15,19].

Another interesting issue is medical care for patients living in nursing homes. Up until now, medical care for patients in nursing homes has usually been carried out by the patients' individual family doctors without a fixed framework of cooperation with the nursing homes and without regular qualification in palliative care on the part of the doctors. The introduction of specialized nursing home physicians may be considered as an alternative. In our survey, this measure (item 14) was predominantly evaluated positively (80% "good") while the feasibility in Germany was evaluated negatively (22% "good").

It is worth mentioning that the medical associations agreed significantly less with the concept of nursing home physicians (65% "good") than did nursing organizations (100% "good") and patient organizations (96% "good"). The negative attitude on the part of the doctors' representatives found in our study is confirmed by recent official statements, e.g. by the German Medical Association: it is argued that nursing home physicians would not solve the main problem of the inappropriately low financing of the time-consuming medical care for nursing home residents [20]. However, with the recent nursing care insurance reform in 2008, the spectrum of possible improvement measures for medical care in nursing homes, which range from formal cooperation with family doctors and specialists to the appointment of nursing home physicians, was extended [21]. One may wonder whether these approaches will actually be made use of.

Also with the recent nursing care insurance reform, a legal right to six months' unpaid leave from work was introduced in Germany for the first time, if employees want to care for their relatives [21]. This measure (item 5) was favoured by nearly all of the participants in our study (92% "good"), but many of them considered the feasibility as being critical (25% "good"). Maybe they are afraid that barriers on the part of employers and employees (e.g. career disadvantages, and in the case of temporary work contracts) will impede realization in practice.

Case management performed by specialized case managers (item 15) tended to be assessed negatively, in particular by the medical associations' representatives. This is not surprising, as many physicians see it as part of their role to coordinate the health care of their patients [11]. However, shortcomings in coordination are among the most serious problems in Germany [19]. There is therefore no doubt that improvements in coordination and management are necessary; but the crucial question is: who is the most appropriate case manager and what form should the management take?

The literature shows that intensive home-based case management provided by registered nurse case managers may, in coordination with patients' existing sources of medical care, improve the realization of care of chronically severely ill patients in the last years of life [22]. Due to the traditional hierarchical structure and the significant cultural and educational barriers between physicians and nurses, it may be presumed that the acceptance of nurses as case managers on the part of the physicians will be difficult to achieve. However, nursing in Germany – as well as in many other countries – has been increasingly professionalized, specialized and academically qualified along with the achievement of new self-confidence [12].

In its recent report, the Advisory Council on the Assessment of Developments in the Health Care System (SVR) points out the need for role changes and new forms of cooperation among health professionals [23]. It is promising that the organization and management of specialized outpatient palliative care was explicitly implemented in the recent German health care reform in 2007 [10,11]. It is greatly to be hoped that appreciable improvements for the patients will result from the political efforts.

### Limitations

There is one powerful lobby group not included in the study: the pharmaceutical industry. This plays an important role in the decision-making and policy-making processes for medical care, e.g. concerning the prices, distribution and availability of drugs within the system of the social health insurance that is responsible for approximately 90% of the citizens in Germany. It was discussed in our study group whether representatives from the pharmaceutical industry should also be surveyed, but we decided against it; we do not think that the industrial perspective should be part of the development of public health targets for palliative care.

Furthermore, it has to be considered that the results are not based on official statements from the institutions, organizations and associations, but reflect the personal attitudes and opinions of the representatives in combination with their professional background, experience and responsibility. We received many emails and telephone calls from participants who wondered if they should take part in the survey as they did not feel adequately qualified to evaluate the specific measures due to a lack of professional focus on palliative care. However, we encouraged them to participate, because it was important for us not to exclusively study experts' opinions. Rather, it was important for us to gain insight into the views of decision-makers and representatives from a wide range of socially and politically relevant groups. Also, we are convinced that a broad approach is needed for the further development of palliative care; that end-of-life questions affect everyone irrespective of their profession; that the professional background influences personal views and vice versa; and that personal experiences and attitudes play an important role in policy-making and decision-making.

## Conclusion

If palliative care is to be improved in Germany, there are significant barriers to be overcome. Substantial improvement measures seem to be considered difficult to implement, possibly because of the traditional fragmentation of responsibilities and the partly conflicting interests of the different stakeholders. The recently initiated national *charter process for the care of very severely ill and dying people *is a promising approach to the further development of palliative care on a broad social, political and scientific basis. It may be helpful to the process that the basic understanding of the palliative care approach seems to be quite homogenous among many stakeholders. However, the awareness of the aims and content of palliative care should be increased on the part of those who are not specialists in palliative care.

## Competing interests

The authors declare that they have no competing interests.

## Authors' contributions

SLL and MB jointly recruited the participants, developed and tested the questionnaire, performed the statistical analyses and made contributions to the manuscript. SB made contributions to the conception and design of the study, the development of the questionnaire and the statistical analysis of data, and to the manuscript. NSCH conceived the study, supervised the recruitment, the development of the questionnaire and the analyses, interpreted the data and drafted the manuscript. All authors read and approved the final manuscript.

## Pre-publication history

The pre-publication history for this paper can be accessed here:



## Supplementary Material

Additional file 1 **Agreement with the WHO-definition**. The table shows the results of the agreement with aspects of the WHO-definition of palliative care for each group. Click here for file

Additional file 2 **Assessment of 18 improvement measures**. The table shows the assessment of 18 selected improvement measures with regard to their meaningfulness in general and their feasibility in Germany for each group. Click here for file
