# Supplementary material for: "Good idea but not feasible" – the views of decision makers and stakeholders towards strategies for better palliative care in Germany: a representative survey
Source: BMC Palliat Care. 2009 Jul 22;8:10. doi: 10.1186/1472-684X-8-10 (PMC2722585; doi:10.1186/1472-684X-8-10)
Supplement: Additional file 1 — Agreement with the WHO-definition. The table shows the results of the agreement with aspects of the WHO-definition of palliative care for each group. [file 1472-684X-8-10-S1.doc]

| **item** | **Statement** | **Frequencies in %*** | | | | | | | | | | | | | | | | | | | | |  |
| --- | --- | --- | --- | --- | --- | --- | --- | --- | --- | --- | --- | --- | --- | --- | --- | --- | --- | --- | --- | --- | --- | --- | --- |
|  | In my opinion it is part of appropriate palliative care to… | all groups** (n=301) | | | patient organizations (n=24) | | | medical associations (n=79) | | | nursing organizations (n=17) | | | health insurance funds (n=55) | | | political institutions (n=24) | | | specialized palliative care (n=39) | | | p |
|  |  | 1 | 2 | 3 | 1 | 2 | 3 | 1 | 2 | 3 | 1 | 2 | 3 | 1 | 2 | 3 | 1 | 2 | 3 | 1 | 2 | 3 |  |
| 1 | …provide relief from pain and other distressing symptoms | 93.3 | 6.0 | 0.7 | 95.8 | 4.2 | 0.0 | 97.4 | 2.6 | 0.0 | 100 | 0.0 | 0.0 | 80.0 | 18.2 | 1.8 | 87.5 | 12.5 | 0.0 | 100 | 0.0 | 0.0 | 0.012¹ |
| 2 | …intend neither to hasten or postpone death | 45.4 | 26.8 | 27.8 | 65.2 | 4.3 | 30.4 | 51.3 | 25.0 | 23.6 | 52.9 | 29.4 | 17.6 | 24.1 | 37.0 | 38.9 | 16.7 | 37.5 | 45.8 | 71.1 | 21.1 | 7.9 | <0.001¹ |
| 3 | …integrate the psychological and spiritual aspects of patient care | 76.0 | 20.7 | 3.4 | 75.0 | 20.8 | 4.2 | 80.8 | 16.7 | 2.6 | 76.5 | 23.5 | 0.0 | 49.1 | 40.0 | 10.9 | 70.8 | 25.0 | 4.2 | 97.4 | 2.6 | 0.0 | <0.001¹ |
| 4 | …enhance quality of life | 79.9 | 16.1 | 3.9 | 91.7 | 4.2 | 4.2 | 80.8 | 14.1 | 5.1 | 76.5 | 23.5 | 0.0 | 63.6 | 25.5 | 10.9 | 73.9 | 26.1 | 0.0 | 89.7 | 0.0 | 10.3 | 0.058 |
| 5 | …offer a support system which helps patients to live as actively as possible until the last moment of life | 60.0 | 23.3 | 16.7 | 75.0 | 12.5 | 12.5 | 67.9 | 15.4 | 16.6 | 58.8 | 17.6 | 23.5 | 40.0 | 45.5 | 14.6 | 58.3 | 29.2 | 12.5 | 71.8 | 17.9 | 10.3 | 0.009¹ |
| 6 | …offer a support system to help the family cope during the patients illness | 50.7 | 23.3 | 26.0 | 66.7 | 8.3 | 25.0 | 64.1 | 19.2 | 16.7 | 64.7 | 23.5 | 11.8 | 20.0 | 30.9 | 49.2 | 41.7 | 20.8 | 37.5 | 79.5 | 15.4 | 5.2 | <0.001¹ |
| 7 | …work multi- and interdisciplinary | 76.3 | 19.3 | 4.4 | 91.7 | 4.2 | 4.2 | 79.5 | 12.8 | 7.7 | 76.5 | 23.5 | 0.0 | 47.3 | 45.5 | 7.2 | 70.8 | 29.2 | 0.0 | 94.9 | 2.7 | 2.6 | <0.001¹ |
| 1=completely agree, 2=agree, 3=undecided, disagree, completely disagree | | | | | | | | | | | | | | | | | | | | | | |  |
| ¹ significant | |  |  |  |  |  |  |  |  |  |  |  |  |  |  |  |  |  |  |  |  |  |  |
| * Percentage may not sum to 100 due to rounding. | | | | | | | | | | | | | | | | | | | | | | |  |
| ** Group "others" is included in the outcome analyses for all groups, but not in the comparison of the groups. | | | | | | | | | | | | | |  |  |  |  |  |  |  |  |  |  |
