# Supplementary material for: "Good idea but not feasible" – the views of decision makers and stakeholders towards strategies for better palliative care in Germany: a representative survey
Source: BMC Palliat Care. 2009 Jul 22;8:10. doi: 10.1186/1472-684X-8-10 (PMC2722585; doi:10.1186/1472-684X-8-10)
Supplement: Additional file 2 — Assessment of 18 improvement measures. The table shows the assessment of 18 selected improvement measures with regard to their meaningfulness in general and their feasibility in Germany for each group. [file 1472-684X-8-10-S2.doc]

| **item** | **statement** |  | **Frequencies of answer "good" in %** | | | | | | |  |
| --- | --- | --- | --- | --- | --- | --- | --- | --- | --- | --- |
|  |  |  | all groups** (n=301) | patient organizations (n=24) | medical associations (n=79) | nursing organizations (n=17) | health insurance funds (n=55) | political institutions (n=24) | specialized palliative care (n=39) | p |
| 1 | Education in palliative care for all involved in professional care is mandatory. | MF* | 97.6 | 100 | 94.8 | 94.1 | 100 | 95.7 | 97.4 | _0.50 |
|  |  | FB | 52.9 | 33.3 | 43.1 | 43.8 | 63 | 59.1 | 54.3 | _0.13 |
| 2 | Compulsory education in palliative medicine for medical students. | MF | 94.3 | 100 | 89.6 | 70.6 | 98.1 | 91.7 | 100 | <0.001¹ |
|  |  | FB | 49.5 | 38.1 | 47.9 | 21.4 | 62.3 | 38.1 | 37.8 | _0.048¹ |
| 3 | Compulsory palliative care training for nurses. | MF | 92.9 | 100 | 86.7 | 82.4 | 94.4 | 87 | 100 | _0.04¹ |
|  |  | FB | 62.6 | 38.1 | 56.9 | 50 | 67.9 | 57.1 | 72.2 | _0.12 |
| 4 | Compulsory training for volunteers in specialized palliative care institution. | MF | 78.1 | 91.7 | 75 | 82.4 | 77.8 | 75 | 84.6 | _0.52 |
|  |  | FB | 46.7 | 50 | 42.4 | 53.3 | 40.4 | 50 | 58.3 | _0.59 |
| 5 | Family members can make use of caring-time (release from work for e.g. 3 months to care for a relative) | MF | 92.3 | 100 | 92.2 | 88.2 | 85.5 | 87.5 | 97.4 | _0.20 |
|  |  | FB | 24.7 | 13 | 23.6 | 17.6 | 25.9 | 34.8 | 22.2 | _0.62 |
| 6 | Employees are protected against dismissal during the caring-time and the following 4 weeks. | MF | 90.6 | 95.7 | 89.6 | 88.2 | 89.1 | 87.5 | 89.7 | _0.96 |
|  |  | FB | 27.9 | 18.2 | 27.9 | 18.8 | 29.6 | 39.1 | 25.7 | _0.65 |
| 7 | Employees get their salary for further 4 more weeks from their employers during the caring-time for dying family members. | MF | 71.6 | 78.3 | 64 | 68.8 | 63 | 65.2 | 84.6 | _0.20 |
|  |  | FB | 13.4 | 0 | 17.4 | 13.3 | 9.3 | 18.2 | 5.6 | _0.18 |
| 8 | Caring-time is not only possible for family-members but also to other people being close to the patient. | MF | 77.5 | 87 | 60.5 | 88.2 | 77.8 | 75 | 92.3 | _0.002¹ |
|  |  | FB | 10.8 | 4.5 | 8.6 | 18.8 | 7.5 | 8.7 | 8.3 | _0.76 |
| 9 | National report about the development of palliative care on an annual basis. | MF | 85.9 | 95.7 | 85.1 | 94.1 | 81.8 | 77.3 | 89.7 | _0.37 |
|  |  | FB | 60.8 | 68.2 | 53.7 | 53.8 | 62.3 | 61.9 | 50 | _0.71 |
| 10 | Development of national goals to improve palliative care involving all relevant social groups. | MF | 87.8 | 100 | 89.5 | 87.5 | 72.7 | 95.8 | 89.7 | _0.007¹ |
|  |  | FB | 48.3 | 42.9 | 52.2 | 50 | 45.3 | 45.8 | 41.2 | _0.92 |
| 11 | Availability of 24-7 regional palliative care services. | MF | 85.8 | 100 | 83.8 | 94.1 | 76.4 | 65.2 | 100 | <0.001¹ |
|  |  | FB | 41.3 | 47.4 | 37.7 | 46.7 | 44.4 | 31.8 | 45.7 | _0.83 |
| 12 | Palliative care patients are completely exempted from additional payments for drugs, cure and aids. | MF | 60.6 | 86.4 | 49.3 | 93.3 | 46.3 | 43.5 | 71.1 | <0.001¹ |
|  |  | FB | 30.6 | 44.4 | 30 | 33.3 | 36.5 | 33.3 | 20 | _0.51 |
| 13 | Availabilty of palliative care teams at every acute care hospital. | MF | 83.2 | 91.7 | 74.7 | 94.1 | 72.7 | 82.6 | 89.2 | _0.09 |
|  |  | FB | 36.7 | 31.6 | 33.8 | 31.2 | 37.7 | 36.4 | 20 | _0.64 |
| 14 | Regular availabilty of nursing home physicians with spezialised training in palliative care. | MF | 79.5 | 95.7 | 65.3 | 100 | 81.8 | 69.6 | 89.7 | <0.001¹ |
|  |  | FB | 21.5 | 15.8 | 20.3 | 12.5 | 25.9 | 19 | 14.3 | _0.75 |
| 15 | Case managers organize and coordinate the care of palliative care patients. | MF | 41.3 | 57.1 | 28.8 | 75 | 38.9 | 36.4 | 41 | _0.012¹ |
|  |  | FB | 23.2 | 33.3 | 13.8 | 14.3 | 28.3 | 38.1 | 16.7 | _0.10 |
| 16 | Telephone hotline for spezialised palliative care advice is available for general practitioners. | MF | 85.6 | 90.9 | 81.6 | 100 | 78.2 | 82.6 | 92.1 | _0.17 |
|  |  | FB | 48.5 | 44.4 | 47.8 | 66.7 | 51.9 | 47.6 | 48.6 | _0.83 |
| 17 | Establishment of publicly funded academic centres for research in palliative care. | MF | 75.1 | 87 | 61.6 | 82.4 | 64.8 | 82.6 | 89.5 | _0.007¹ |
|  |  | FB | 29.5 | 23.5 | 29 | 35.7 | 21.2 | 28.6 | 31.4 | _0.85 |
| 18 | Public information campaigns in palliative care issues. | MF | 85.4 | 91.7 | 81.6 | 94.1 | 83.3 | 87 | 89.7 | _0.62 |
|  |  | FB | 62.2 | 66.7 | 65.2 | 71.4 | 58.5 | 63.6 | 50 | _0.63 |
| ¹ significant | |  |  |  |  |  |  |  |  |  |
| * MF = meaningfulness; FB = feasibility | |  |  |  |  |  |  |  |  |  |
| ** Group "others" is included in the outcome analyses for all groups, but not in the comparison of the groups. | | | | | | |  |  |  |  |
